# *Cyclocarya paliurus* extract activates insulin signaling via Sirtuin1 in C2C12 myotubes and decreases blood glucose level in mice with impaired insulin secretion

**DOI:** 10.1371/journal.pone.0183988

**Published:** 2017-08-31

**Authors:** Hisae Yoshitomi, Rika Tsuru, Linyi Li, Jingxin Zhou, Maya Kudo, Tonghua Liu, Ming Gao

**Affiliations:** 1 School of Pharmaceutical Sciences, Mukogawa Women’s University, Hyogo, Japan; 2 Health-cultivation Laboratory of the Ministry of Education, Beijing University of Chinese Medicine, Beijing, China; 3 Dongzhimen Hospital Eastern Affiliated to Beijing University of Chinese Medicine, Beijing, China; Universidad Pablo de Olavide, SPAIN

## Abstract

Diabetes is caused by the lack of release or action of insulin. Some foods and supplements can compensate for this deficiency; thus, they can aid in the prevention or treatment of diabetes. The aim of this study was to investigate the effects of Cyclocarya paliurus extract (CPE) on insulin signaling and its capacity to correct hyperglycemia in the absence of insulin. To investigate the hypoglycemic effects of CPE, C2C12 cells were exposed to CPE (50 and 100 μg/mL). CPE promoted 2-(N-(7-Nitrobenz-2-oxa-1,3-diazol-4-yl)Amino)-2-Deoxyglucose (2NBDG) uptake into the cells via translocation of glucose transporter 4 (Glut4) to the plasma membrane. In addition, CPE enhanced tyrosine phosphorylation of insulin receptor substrate and activated phosphatidylinositol 3-kinase and protein kinase B (Akt) via sirtuin1 in C2C12 cells. Moreover, we found that oral administration of CPE (1 g/kg) to streptozotocin-induced hyperglycemic mice produced a progressive decrease in plasma glucose levels at 1 h after single dosing. At that point, CPE significantly increased the expression of skeletal muscle membrane Glut4 and enhanced the phosphorylation of Akt. These results suggest that CPE exerts antidiabetic effects similar to those of insulin, and may be an oral therapeutic alternative for the management of diabetes.

## Introduction

Diabetes mellitus, one of the most common chronic diseases, results from reduced insulin secretion or insulin response in the peripheral tissues. It has been shown that long-term hyperglycemia can lead to coronary artery disease and cerebrovascular disease [1]. The total number of diabetic patients is estimated to rise to 300 million worldwide by 2025 according to the World Health Organization (WHO) [2]. Thus, the control of blood glucose level is of vital importance.

Insulin is an essential hormone for maintenance of glucose homeostasis. Its binding to insulin receptors (IRs) promotes the insulin receptor substrates (IRS)-phosphatidylinositol 3-kinase (PI3K)-protein kinase B (Akt) pathway [3]. Finally, activation of Akt decreases the circulating glucose levels via suppression of glucose production by the liver and promotion of glucose uptake by adipose tissues and skeletal muscles.

Insulin is formulated as pharmaceutical products, particularly for management of hyperglycemia owing to imperfect insulin secretion. However, insulin is a peptide with molecular weight more than 5000 Da and rapidly degraded at the digestive tract; thus, it is only administered by injection. In addition, the other therapeutic agents, such as sulfonylureas and glinides, promote insulin secretion by the pancreatic β-cells, which result in side effects, such as hypoglycemia, weight gain, and pancreatic β-cells exhaustion. Therefore, the development of oral agents with insulin-like action and good safety profile to control the blood glucose level is needed in the fields of pharmaceutical products and food science.

Cyclocarya paliurus (Batal.), which belongs to the genus Cyclocarya (Juglandaceae), has beneficial effects on health. C. paliurus is known to prolong lifespan without any adverse effects or toxicity [4]. An epidemiological study showed that hyperglycemia and diabetes mellitus are very rare in Xiushui area in China owing to the benefits of C. paliurus in prevention of these diseases [5, 6]. Previous studies showed that C. paliurus aqueous extract inhibited α-glucosidase activity and decreased blood glucose level in diabetic mice [7]. In addition, Wang et al. evaluated the antihyperglycemic effect of the ethanol and aqueous extracts of C. paliurus in high fat diet- and streptozotocin-induced diabetic rats [8]. However, the underlying mechanisms are still not fully elucidated.

In the present study, we showed the effects of *C*. *paliurus* extract (CPE) on glucose transportation into cell without insulin stimulation using C2C12 myotube cells. Moreover, we investigated the detailed mechanisms, including insulin signaling and Sirtuin1 (Sirt1). In addition, we determined that effects of oral administration of CPE to streptozotocin (STZ)-induced insulin deficient hyperglycemic mice after single dosing whether CPE possesses an insulin-like action.

## Materials and methods

### Preparation of extracts

Extracts of C. paliurus were provided from Beijing University of Chinese Medicine. The dry leaves (10 kg) were extracted with 75% ethanol (EtOH). After the solvent was evaporated under reduced pressure, the residue was suspended in water (10 L) and partitioned with petroleum ether, chloroform, and ethyl acetate (EtOAc), successively. The aqueous layer was evaporated under reduced pressure, then subjected to column chromatography on macroporous adsorption resin (HP-20), and continuously eluted with H2O (20 L) and 30% EtOH (20 L). The ethanolic part represented CPE (147 g). The concentration of chlorogenic acid, cryptochlorogenic acid and quercetin-3-O-β-D-glucuronide in CPE were determined to be 1.83%, 1.02% and 29.12%, respectively, using high-performance liquid chromatography (HPLC) analysis (S1 Text and S1–S3 Figs).

### C2C12 cell differentiation and treatment

C2C12 cells were maintained in low-glucose Dulbecco's modified Eagle's medium (DMEM) with 10% fetal bovine serum (FBS). Cells were obtained by culturing myoblasts in DMEM containing 2% horse serum for at least 5 days. After differentiation, cells were treated with CPE (50 or 100 μg/mL) or insulin (100 nM) in serum-free medium for 30 min. Cells were pretreated 30 mins with inhibitors, 100 μM HNMPA(AM)_3_ (Santa Cruz Biotechnology, Inc.) or 40 μM EX527 (BPS Bioscience, USA) before CPE treatment.

### Cell viability assay

Cell proliferation was assessed using the neutral red uptake assay, as previously described [9]. Briefly, after exposure to CPE, cells were incubated at 37°C for 3 h in a medium supplemented with neutral red (50 μg/mL) and subjected to a mixture of acetic acid (1%) and EtOH (50%) to extract the dye. The absorbance of each well was measured at 540 nm using a microplate reader.

### Measurement of 2NBDG uptake

Differentiated C2C12 cells were starved in a serum-free DMEM for 3 h before treatment with the drugs. After incubation, C2C12 cells were incubated with CPE or insulin in glucose-free DMEM for 1.5 h. After incubation, the cells were washed with PBS and then incubated in fresh glucose-free DMEM containing 100 μM 2NBDG for 30 mins. After washing by PBS, cells were measured using a fluorescence microplate reader at excitation and emission wavelengths of 485 and 530 nm. Nonspecific uptake determined in the presence of 10μM cytochalasin B (Nacalai Tesque) and background fluorescence of CPE were subtracted from all values. Cells were treated with inhibitors, 100 μM HNMPA(AM)_3_, 40 μM EX527 and 50 μM Perifosine (Cayman chemical, USA). In addition, images of the cells were captured using a fluorescence microscope (Olympus, Japan).

### Extraction of cytosol and membrane protein complexes

The cells and isolated tissues were homogenized in 200 μL of Tris-buffered saline (TBS) (0.025 M Tris-HCl, 0.15 M NaCl, pH 7.2) and centrifuged (1000 g, 5 min, 4°C). The cytosol fraction and membrane protein was extracted using Mem-PER eukaryotic membrane protein extraction kit (PromoKine, UK). Recovered proteins were mixed with SDS buffer, boiled for 5 min, and loaded on SDS-PAGE. Markers of purity and normalization for cytosolic and membrane protein fractions were used by GAPDH and Na/K ATPase.

### Primary and secondary antibodies

Immunoblotting was performed with the following commercially available antibodies: AS160, phospho-AS160, Akt, phospho-Akt, IRS-1, phospho-IRS-1 (Ser307, Ser612, Ser1101), AMPK, phospho-AMPK, Na/K ATPase, GAPDH, anti-goat IgG, anti-rabbit IgG, and anti-mouse IgG from Cell Signaling Technology (Beverly, MA, USA). Phospho-IRS-1 (Tyr 989), and PI3-kinase p85α, Tyrosine were from Santa Cruz Biotechnology. β-actin was obtained from Sigma (St. Louis, Mo, USA).

### Western blotting

The protein was extracted via homogenization in an ice-cold RIPA buffer. Equal amounts of the proteins were electrophoresed and transferred onto a polyvinylidene difluoride (PVDF) membrane. The membrane was blocked, and the blot was incubated successively with the primary and secondary antibodies. Finally, detection was achieved using Chemi-Lumi one super (Nacalai Tesque, Kyoto). The density of the bands was measured using Image J public domain software from the National Institutes of Health.

### Immunoprecipitation

Immunoprecipitation was performed according to a previously described method [10]. Briefly, protein extracts were immunoprecipitated with gentle rotation in the presence of the desired antibody for 4 h at 4°C. After incubation with Protein A/G PLUS-Agarose (Santa Cruz Biotechnology, Inc.) overnight at 4°C with mixing, the antigen-antibody complexes were precipitated by brief centrifugation. Then, the pellets were washed in RIPA buffer and used for western blotting.

### Sirt1 activity

Sirt1 activity was measured by Sirt1 activity assay kit according to the manufacturer’s instructions (Cayman chemical USA). Human recombinant Sirt1 was incubated with CPE (0.5, 2.5, and 5 μg/mL) or resveratrol (5 μM), a Sirt1 activator, as well as substrate peptide, nicotinamide adenine dinucleotide (NAD), and developer. The fluorescence intensity was measured using excitation and emission wavelengths of 360 and 460 nm, respectively. Results were expressed as percentage of the control.

### Sirt1 siRNA transfection

siRNA transfection was performed using the GenMute^™^ siRNA Transfection Reagent for C2C12 Cell (SignaGen Laboratories, USA) according to the manufacturer’s protocol. Briefly, it was carried out before 60% cell confluency. Transfection reagents and Sirt1 siRNA or scrambled siRNA (Santa cruz, CA) as a control were individually diluted and mixed. Mixtures were added to each well and incubated for 18 h. Then, media were removed and replaced with an induction medium. The knockdown of Sirt1 was confirmed by immunoblotting.

### Animals

Six-week-old male specific pathogen-free (SPF) ICR mice were supplied by Japan SLC (Shizuoka, Japan). All mice were housed in a climate-controlled (temperature; 22~24°C, humidity; 40~60%) light-regulated room under 12-hour light and dark cycles. All mice received normal chow (CE-2) with water *ad libitum* during the experimental period. All procedures were carried out in accordance with the guiding principles of the care and use of animals in the field of physiological sciences established by the Physiological Society of Japan. The study was approved by the ethics committee of Laboratory Animals at Mukogawa Women’s University (P-06-2014-02-A).

### Induction of diabetes in mice and treatment

After an adaptation period of 1week, ICR mice were injected intraperitoneally with a single dose of STZ (200 mg/kg) dissolved immediately in saline. Mice injected with an equal volume of saline were maintained as normal groups. Body Weights, food and water intake were recorded every day. 4-days after the injection of STZ or saline, blood glucose levels were assessed using a Glutest censor (SKK, Japan). When the blood glucose levels of STZ-treated mice were > 300 mg/dL, these mice were diagnosed as diabetic. Then, these mice were randomly divided into 2 groups, a control group (n = 8) and a CPE group (n = 9). Body weight and glucose level did not have a difference between control group and CPE group (Table 1). i) For determination of blood glucose and insulin levels, blood samples were withdrawn at 0, 1, and 2 h after oral administration of CPE (1 g/kg). ii) One hour after administration of CPE, the mice were euthanized by an overdose of sodium pentobarbital (65 mg/kg i.p.). Blood was collected by cardiac puncture and muscles were isolated immediately. All efforts were made to minimize suffering.

**Table 1:** General characteristics of ICR mice used in the study.

	STZ		Normal (n = 5)
	Control (n = 8)	CPE (n = 9)	
Body Weight (g)	42.9 ± 0.7	41.6 ± 0.6	42.0 ± 0.4
Blood Glucose (mg/dL)	426.8 ± 17.1	418.9 ± 11.3	121.0 ± 9.1**
Food intake (g/day)	10.3 ± 0.5	9.8 ± 0.5	4.8 ± 0.4**
Water intake (mL/day)	39.3 ± 4.0	40.5 ± 5.5	8.3 ± 1.0**

Data are expressed as the mean ± SEM.

**p < 0.01 *vs*. the control group.

### Statistical analysis

Data were expressed as the mean ± standard error of the mean (SEM) of three independent experiments. Statistical analysis of data was performed using Student’s t-test and one-way analysis of variance followed by Dunnett’s or Tukey's test to determine the significance of the difference. A p-value < 0.05 was considered statistically significant.

## Results

### CPE promoted glucose uptake *via* stimulation of Glut4 translocation in C2C12 cells in an insulin-independent manner

**Fig 1:**
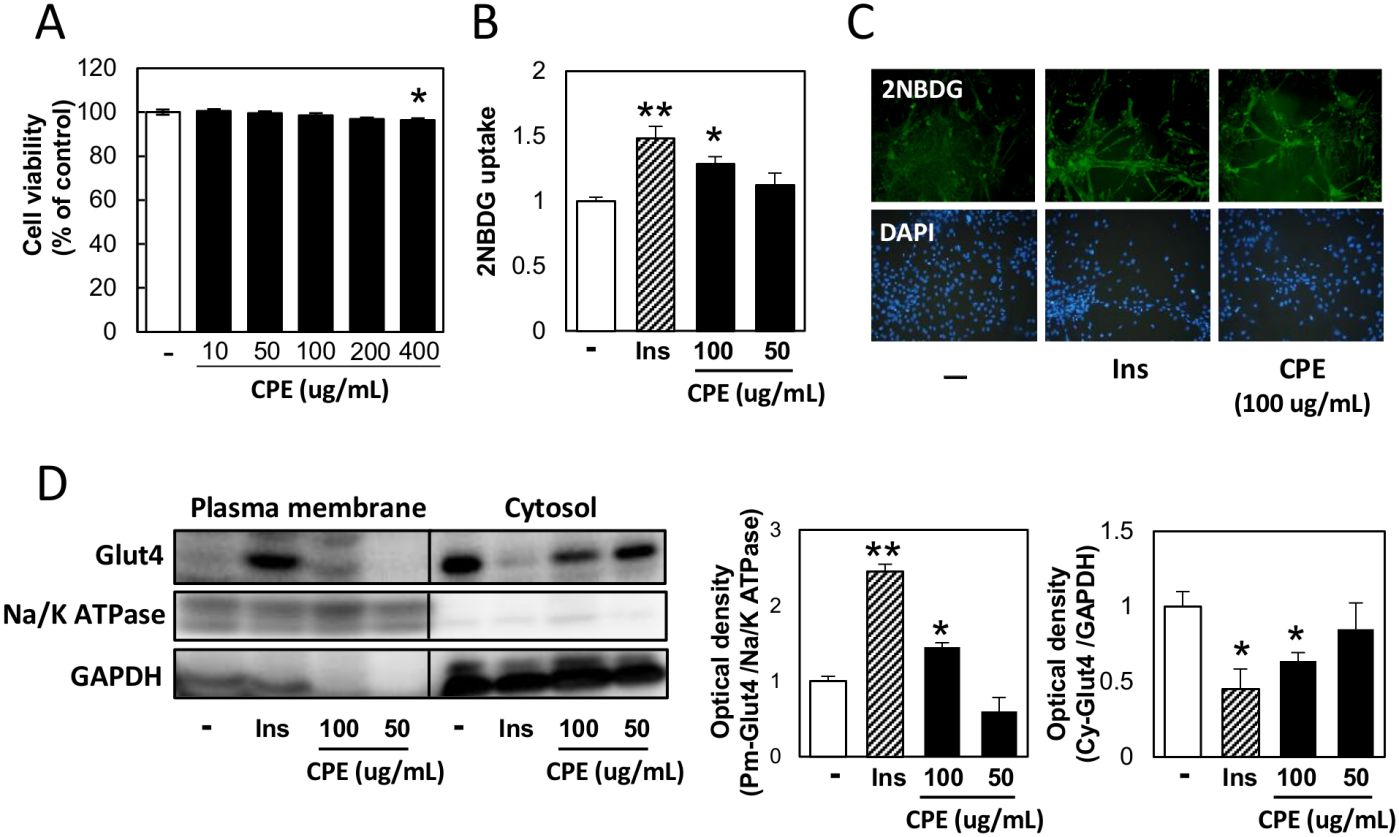
CPE promotes glucose uptake in C2C12 cells. C2C12 cells were treated with CPE (0–400 μg/mL) for 3 h, and cell viability was measured by neutral red (A). 2NBDG uptake by the cells exposed to CPE (50 or 100 μg/mL) or insulin (100 nM) for 1.5 h was measured using a fluorescence microplate reader (B) or microscopy (C). Effect of CPE on Glut4 translocation; plasma membrane fraction (Pm-Glut4), cytosol fraction (Cy-Glut4). Glut4 expression levels in the each fraction were normalized by Na/K ATPase and GAPDH (D). Data are expressed as the mean ± SEM of three independent experiments. *p < 0.05, **p < 0.01 *vs*. the vehicle-treated control.

The two concentrations (50 and 100 μg/mL) were selected by cell viability assay (Fig 1A). To investigate the effects of CPE on glucose uptake, we performed a glucose-uptake assay using a fluorescent D-glucose derivative, 2NBDG in C2C12 cells. 100 μg/mL CPE significantly increased glucose uptake, compared to the control group (Fig 1B). As shown in Fig 1C, fluorescence microscopy showed increased uptake in the 100 μg/mL CPE-exposed group. Translocation of Glut4 to the plasma membrane stimulates the uptake of glucose by the cell. Thus, we determined Glut4 levels in the fractionated plasma membrane and cytoplasm by immunoblotting. 100 μg/mL CPE treatment increased the expression of Glut4 in the plasma membrane, whereas it decreased Glut4 levels in the cytoplasm (Fig 1D).

### CPE activated insulin signaling in C2C12 cells without insulin stimulation

**Fig 2:**
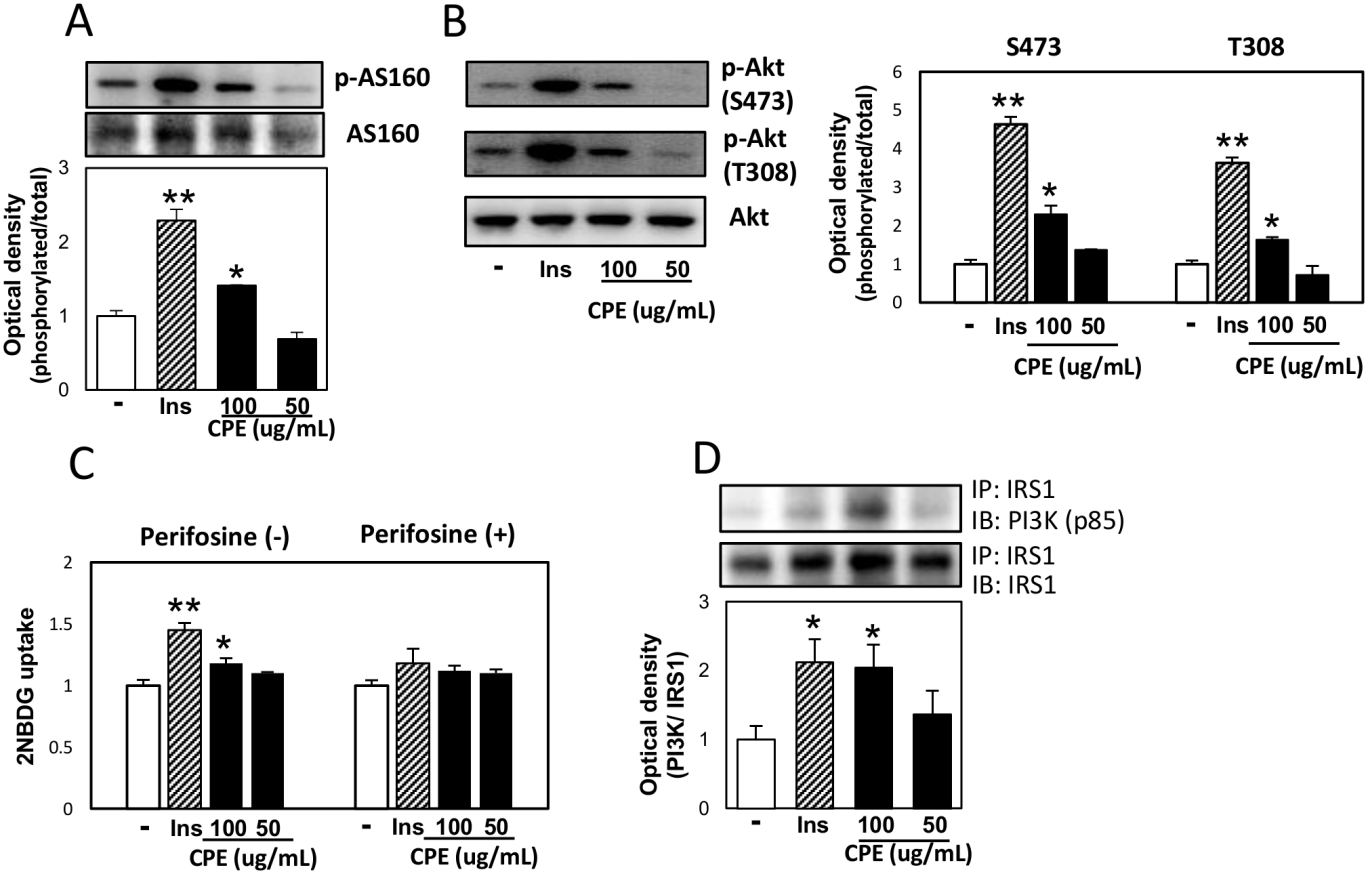
CPE activated PI3K-Akt-AS160 signaling in C2C12 cells. C2C12 cells were exposed to CPE (50 or 100 μg/mL) or insulin (100 nM) for 30 min. The phosphorylation level of AS160 at Thr642 (A), Akt at Ser473 and Thr308 (B), and effects of CPE on the 2NBDG uptake in C2C12 cells treated with 50 μM Perifosine, Akt inhibitor (C). IRS1 and p85 subunit of PI3K complex (D) was determined by immunoprecipitation. Data are expressed as the mean ± SEM of three independent experiments. **p < 0.01 *vs*. the vehicle-treated control.

**Fig 3:**
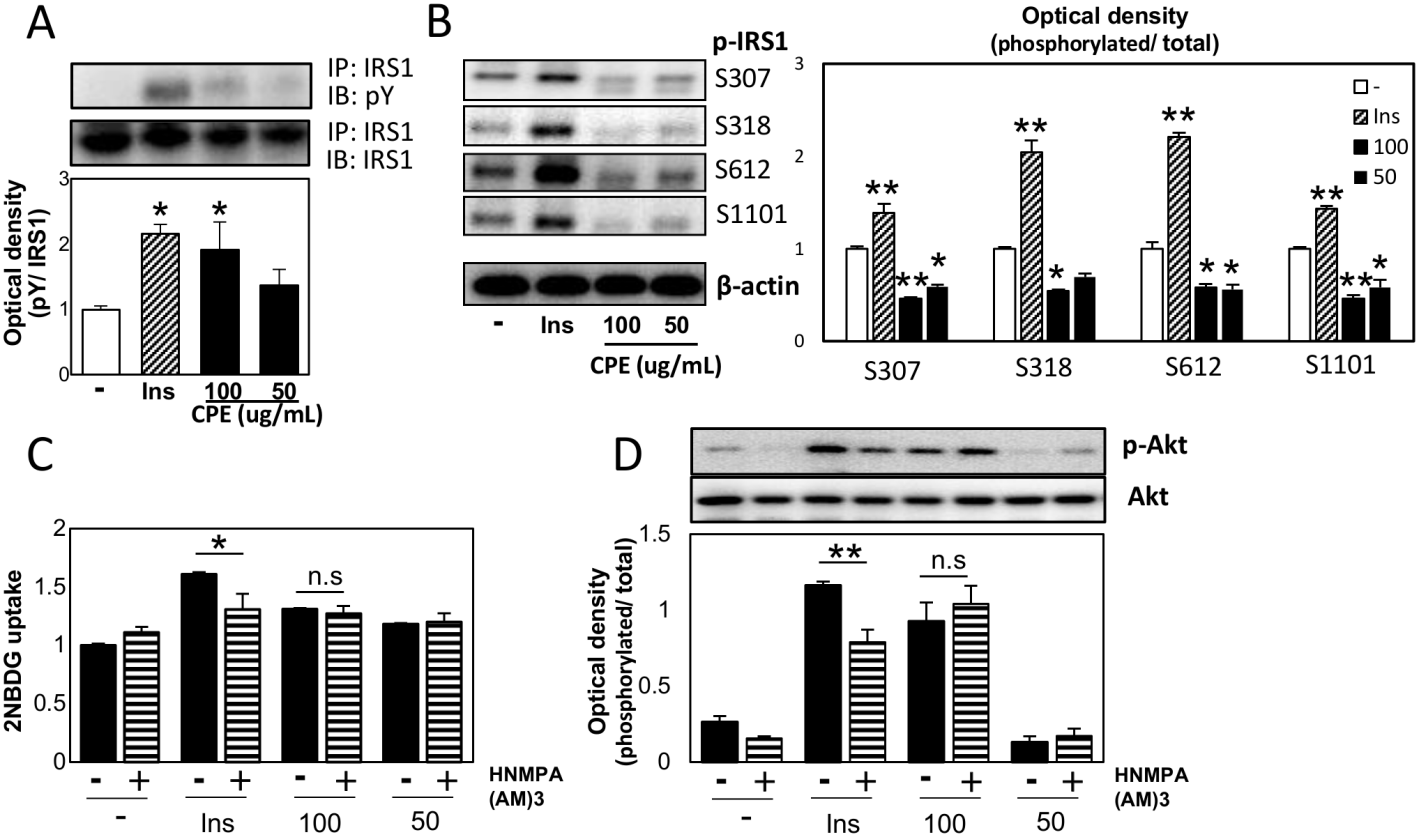
CPE affected IRS1 but not insulin receptor in C2C12 cells. Tyrosine phosphorylation level of IRS1 (A), phosphorylation level of IRS1 at serine307, 318, 612 and 1101 (B) were investigated. Effects of CPE on the 2NBDG uptake (C) and phosphorylation level of Akt (D) in C2C12 cells treated with 100 μM HNMPA(AM)_3_, p-IR inhibitor. Data are expressed as the mean ± SEM of three independent experiments. **p < 0.01 *vs*. the vehicle-treated control.

To elucidate the mechanism of regulation of glucose uptake by CPE, we measured the protein expression of the factors controlling the translocation of Glut4, particularly those involved in insulin signaling in C2C12 cells. As shown in Fig 2A, it was found that the phosphorylation level of AS160, which is located immediately upstream of Glut4 translocation, increased in C2C12 cells with CPE in the absence of insulin. Moreover, phosphorylation of Akt was enhanced by CPE treatment and pre-treatment with the Akt inhibitor, Perifosine resulted in the loss of CPE inducible effect on 2NBDG uptake (Fig 2B and 2C). Furthermore, activation of PI3K was increased after CPE treatment (Fig 2D). IR substrate (IRS) is one of the major substrates of IR kinase [11]. IRS1, located upstream of PI3K, is activated by tyrosine phosphorylation, whereas serine phosphorylation adversely affects its activity. Additionally, CPE treatment enhanced the phosphorylation of tyrosine in IRS-1, and reduced the phosphorylation of ser307, 318, 612, 1101, compared to the control cells (Fig 3A and 3B). Furthermore, pre-treatment with the IR inhibitor, HNMPA(AM)_3_ did not affect CPE-induced 2NBDG uptake and phosphorylation of Akt (Fig 3C and 3D). This suggests that CPE activates insulin signaling cascade independently of IRs.

### CPE affected insulin signaling *via* Sirt1 in C2C12 cells

**Fig 4:**
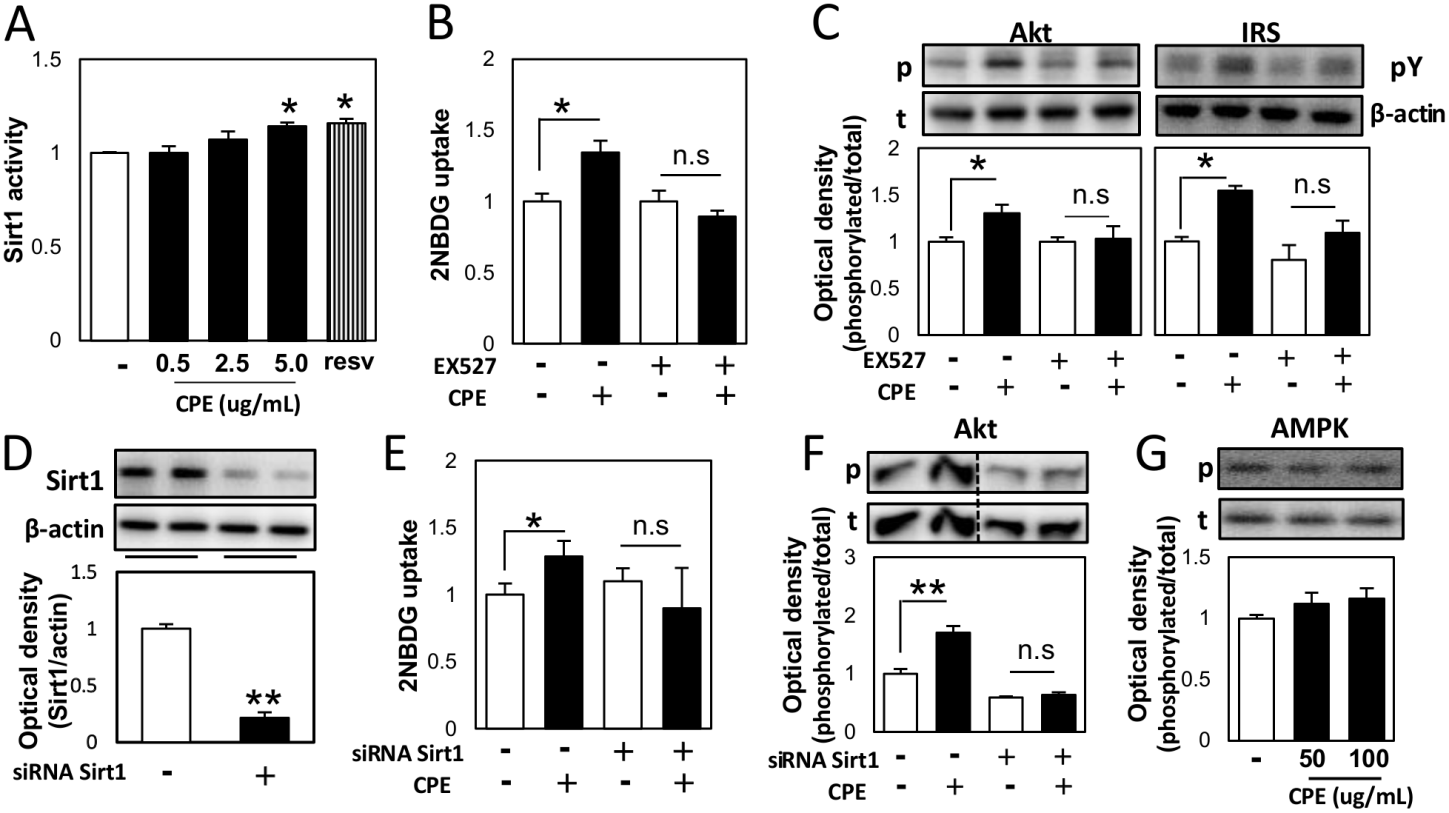
CPE affected insulin signaling *via* Sirt1 in C2C12 cells. Human recombinant Sirt1 were incubated with CPE (0.5, 2.5, and 5 μg/mL) or resveratrol (5 μM), a Sirt1 activator, as well as substrate peptide, NAD, and developer, and Sirt1 activity was measured (A). C2C12 cells were incubated with CPE (100 μg/mL) with or without treatment with EX527 (40 μM), and 2NBDG uptake (B), Akt phosphorylation at Ser473, and tyrosine phosphorylation of IRS (C) were measured. Sirt1 expression was determined after treatment with Sirt1 siRNA (D). Cells were incubated with CPE (100 μg/mL) with or without Sirt1 knockdown, and 2NBDG uptake (E) and Akt phosphorylation level at Ser473 (F) were determined. The effects of CPE on the phosphorylation level of AMPK (G). Values are expressed as the mean ± SEM (*n* = 3–4). *p < 0.05, **p < 0.01 *vs*. the vehicle-treated control.

Some enzymes are known for regulator of insulin signaling pathway [12–14]. Sirt1 is one of the factors involved in regulation of this signaling [15]. CPE activated Sirt1 directly in a concentration-dependent manner (Fig 4A). Furthermore, pre-treatment with the Sirt1 repressor, EX527 resulted in the loss of CPE inducible effect on 2NBDG uptake (Fig 4B). Similarly, the effect of CPE on the phosphorylation of IRS1 tyrosine and Akt was investigated in the presence of EX527. EX527 diminished the effect of CPE on IRS1 tyrosine and Akt phosphorylation (Fig 4C). In addition, Sirt1 knockdown diminished the effect of CPE on 2NBDG uptake and AKT phosphorylation (Fig 4D, 4E and 4F). Sirt1 is known to mediate AMP-activated protein kinase (AMPK) activation. Our results showed that CPE did not have a significant effect on AMPK phosphorylation (Fig 4G).

### Oral administration of CPE decreased blood glucose level in STZ-induced diabetic mice

**Fig 5:**
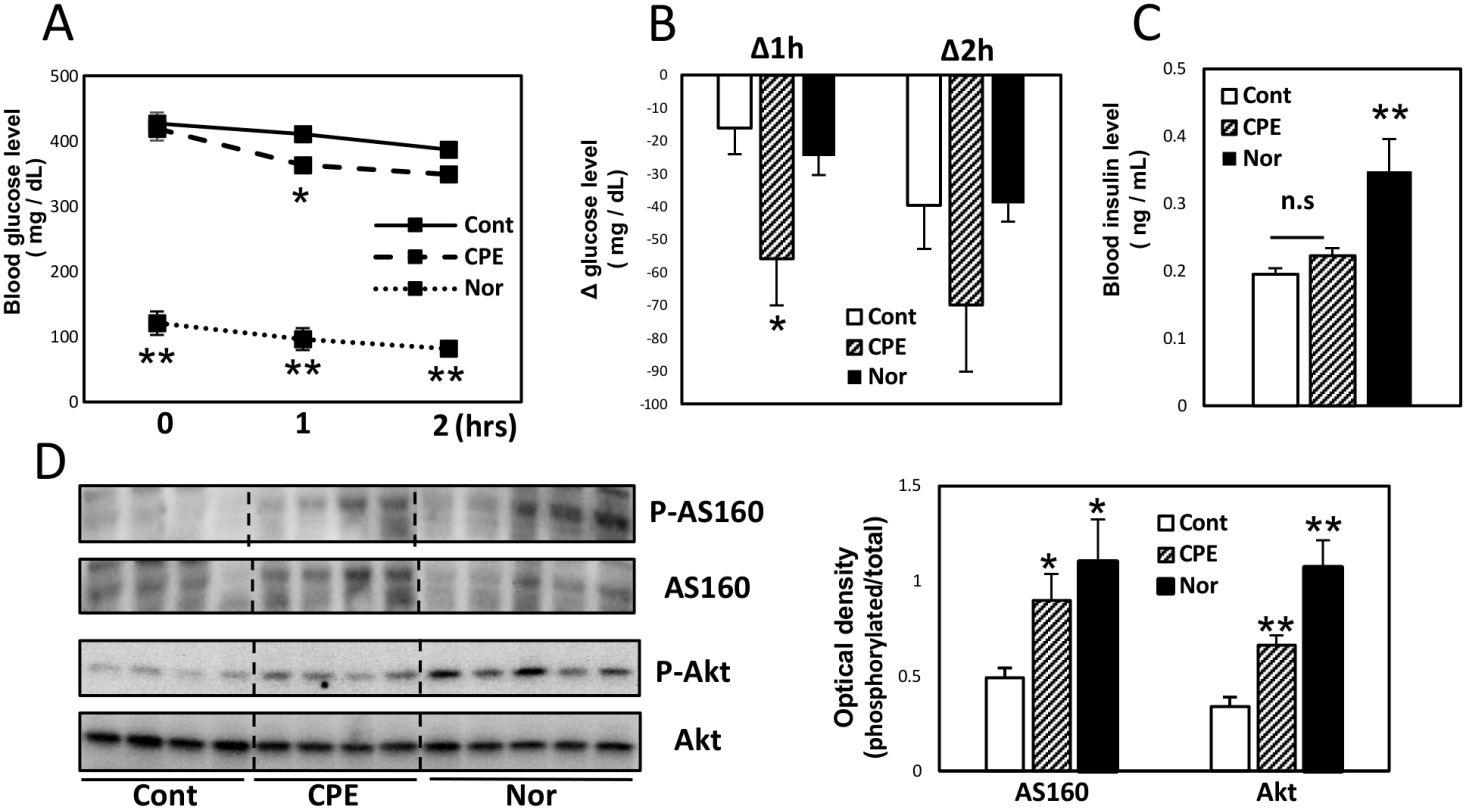
Oral administration of CPE decreased blood glucose level in STZ-induced diabetic mice. ICR mice were injected intraperitoneally with a single dose of STZ (200 mg/kg) and randomly divided into 2 groups, a control group (Cont) and a CPE administration group (CPE). Mice injected with an equal volume of saline were maintained as normal groups (Nor). Blood glucose levels at 1 and 2 h after oral administration of CPE (1 g/kg) to STZ-induced diabetic mice (A), and the change from before administration (B). Blood insulin concentrations (C) and phosphorylation level of AS160 and Akt in the skeletal muscles (D) at 1 h after administration. Data are expressed as the mean ± SEM (*n* = 5–9) *p < 0.05, **p < 0.01 *vs*. the control group.

We expected that CPE might exert an insulin-like effect *in vivo*, thus, we investigated the effects of CPE on blood glucose level in STZ-induced diabetic mice. Oral administration of CPE (1 g/kg) to mice produced a moderate decrease in plasma glucose level at 1 h after single dosing (Fig 5A and 5B). At this time, there was no significant difference in plasma insulin level between the control group and CPE group (Fig 5C). The reduction in blood glucose level induced by CPE treatment in STZ-induced diabetic mice did not correlate with the insulin level. Moreover, CPE treatment significantly increased the phosphorylation of AS160 and Akt in the skeletal muscles, compared to the control group (Fig 5D).

## Discussion

In this study, we found that CPE activated insulin signaling (IRS1-PI3K-Akt-AS160) in an insulin-independent manner, which resulted in an increase in glucose uptake *via* induction of Glut4 translocation in C2C12 cell line. The observed effect was associated with an increase in Sirt1 activity. Moreover, we showed that oral administration of CPE (1 g/kg) to STZ-induced hyperglycemic mice produced a moderate decrease in plasma glucose level at 1 h after single dosing.

Recent studies showed that aqueous extracts of various plants mimicked the effect of insulin on glucose metabolism [16, 17]. In addition, several previous studies have shown the hypoglycemic effect of *C*. *paliurus* and its mechanisms. Kurihara showed that a single administration of CPE reduced sucrose-induced increase in blood glucose in ICR mice, and suggested that CPE controlled glucose absorption *via* α-glucosidase inhibition [7]. Moreover, two triterpenoids, cyclocaric acid B and cyclocarioside H, found in *C*. *paliurus* promoted glucose uptake by the adipose cells, and ameliorated insulin signaling owing to its anti-inflammatory activity *via* induction of AMPK [18]. However, our study showed that the hypoglycemic effects of CPE might be attributed to different mechanisms.

Skeletal muscles account for approximately 75% of glucose uptake under insulin-stimulated conditions [19], thus, they are considered as an important therapeutic target for management of diabetes. The mouse muscle cell line, C2C12 has been widely used to investigate the effect of various agents on glucose transport. Thus, we employed C2C12 myotubes to evaluate the effect of CPE on glucose uptake, and found that CPE treatment promoted glucose uptake and Glut4 translocation from the endoplasmic reticulum to the plasma membrane to regulate glucose uptake.

Insulin signaling pathway is involved in glucose uptake. AS160 regulates insulin-stimulated Glut4 trafficking, wherein its phosphorylation at Thr642 is necessary for Glut4 translocation [20]. Our study showed that CPE induced not only AS160 phosphorylation but also the upstream pathways of AS160, such as IRS1-PI3K-Akt pathway. Interestingly, these effects of CPE were independent of insulin, which supported its role as an insulin mimetic. The action of insulin is initiated by its binding to the IR, which stimulates a complex cascade of downstream insulin signaling [21]. Some compounds, including L-783, 281, and tannic acid, have been shown to act on IR [22, 23]. However, our results suggested that CPE did not affect to IR to stimulate insulin signaling.

Besides regulation of insulin signaling, Sirt1 is involved in the activation of tyrosine phosphorylation and inhibition of serine phosphorylation of IRS1. Phosphorylation of tyrosine in IRS activates PI3K and subsequently phosphorylates Akt [24]. In contrast to the phosphorylation of tyrosine, serine phosphorylation played an important role as a mechanism that negatively regulated insulin signaling [25, 26]. Thereby the activation of tyrosine phosphorylation and inhibition of serine phosphorylation of IRS1 results in activation of Akt in insulin-sensitive cells. Furthermore, Sirt1 positively regulates insulin signaling through deacetylation of IRS2 and activation of Akt [15]. Since Sirt1 can reduce insulin resistance and treat type 2 diabetes [15]. Our findings showed that CPE increased Sirt1 activity. Furthermore, EX527, an inhibitor of Sirt1, diminished the effects of CPE on glucose uptake and phosphorylation of insulin signaling components. These results showed that Sirt1 activation by CPE might have immediate effects on insulin signaling, which promoted glucose uptake. However, the detailed mechanisms are not fully elucidated. We could not emphasize whether Sirt1 regulated insulin signaling directly or not. Further studies are needed to investigate the regulation of insulin signaling by Sirt1.

Recent study has shown that AMPK also control AS160 phosphorylation to induce glucose uptake via Glut4 translocation in an insulin independent manner [27]. Sirt1 activates AMPK through deacetylation and activation LKB1 [28]. In this work, CPE did not impact on AMPK activity.

More importantly, we showed that CPE treatment facilitated the cellular glucose uptake in C2C12 cells *via* translocation of Glut4 the cell membrane, in the absence of insulin. These findings suggested that CPE could be a potential therapy for diabetes, as an oral hypoglycemic drug. Thus, we conducted an additional experiment using STZ-induced Type 1 diabetic mice to confirm the insulin-like action of CPE *in vivo*. We found that the reduction in blood glucose by a single oral dose of CPE did not correlate with the increase in insulin concentration. Moreover, CPE treatment increased the phosphorylation of Akt and AS160 in both the i*n vivo* and *in vitro* experiments.

Approximately 50 compounds have been isolated from *C*. *paliurus*, including flavonoids, polysaccharides, triterpenoids, carbohydrates, and sterols [29, 30]. However, the main active compound(s) responsible for the beneficial effects of CPE are not known. Componential analysis of *C*. *paliurus* requires further study.

Taken together, this study showed that CPE exerted an acute insulin-like antihyperglycemic effect in STZ-induced diabetic mice. The mechanism of action of CPE might involve stimulation of Sirt1 and promotion of insulin signaling pathway. The insulin-like action of CPE might help to correct the diabetics and decrease the required insulin injections. Therefore, CPE represents a possible dietary adjunct for treatment of diabetes and a potential source of new orally active agents for future diabetes therapy.

## Supporting information

S1 Fig Typical chromatograms for the determination of chlorogenic acid in CPE. (A) Chromatogram of the reference standards of chlorogenic acid. (B) Chromatogram of the CPE extract.
(TIF)

S2 Fig Typical chromatograms for the determination of cryptochlorogenic acid in CPE. (A) Chromatogram of the reference standards of cryptochlorogenic acid. (B) Chromatogram of the CPE extract.
(TIF)

S3 Fig Typical chromatograms for the determination of quercetin-3-O-β-D-glucuronide in CPE. (A) Chromatogram of the reference standards of quercetin-3-O-β-D-glucuronide. (B) Chromatogram of the CPE extract.
(TIF)

S1 Text High-performance liquid chromatography (HPLC) analysis method. (DOCX)
