# Supplementary figures and images for: Cyclocarya paliurus extract activates insulin signaling via Sirtuin1 in C2C12 myotubes and decreases blood glucose level in mice with impaired insulin secretion
Source: PLoS One. 2017 Aug 31;12(8):e0183988. doi: 10.1371/journal.pone.0183988 (PMC5578601; doi:10.1371/journal.pone.0183988)

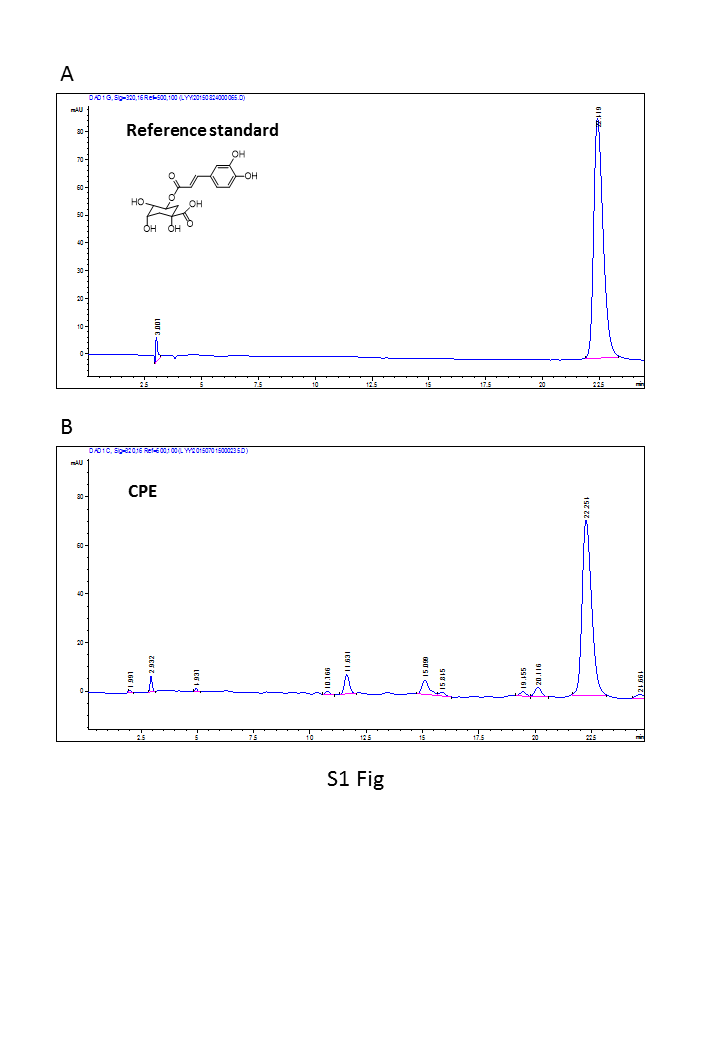

Supplement: S1 Fig — (A) Chromatogram of the reference standards of chlorogenic acid. (B) Chromatogram of the CPE extract. (TIF) [file pone.0183988.s001.TIF]

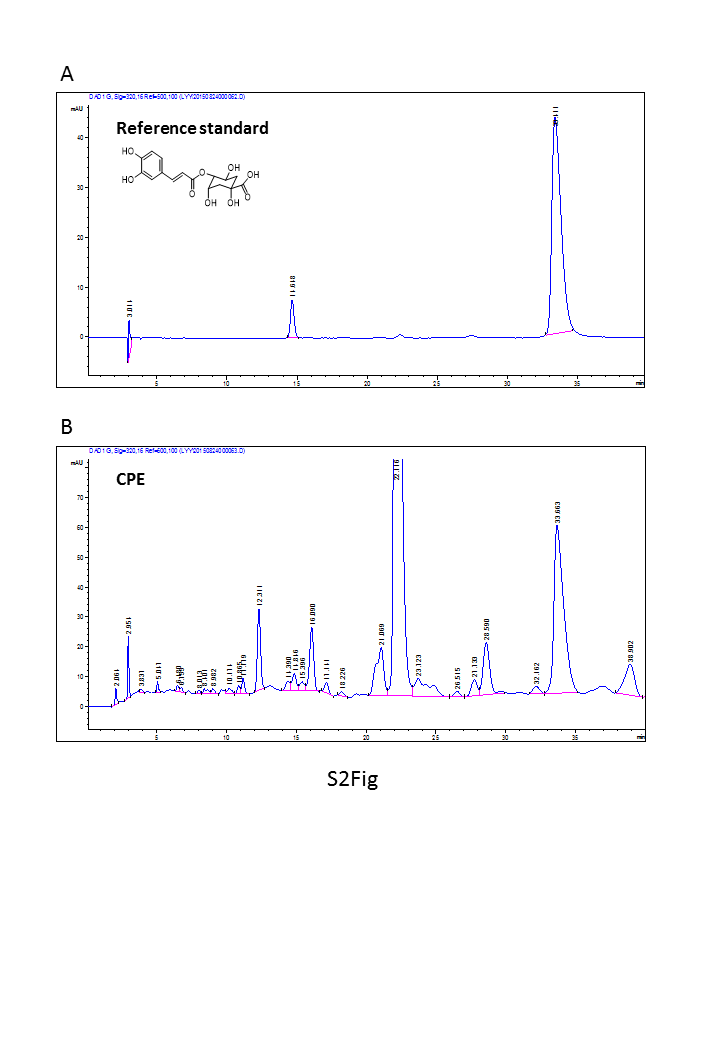

Supplement: S2 Fig — (A) Chromatogram of the reference standards of cryptochlorogenic acid. (B) Chromatogram of the CPE extract. (TIF) [file pone.0183988.s002.tif]

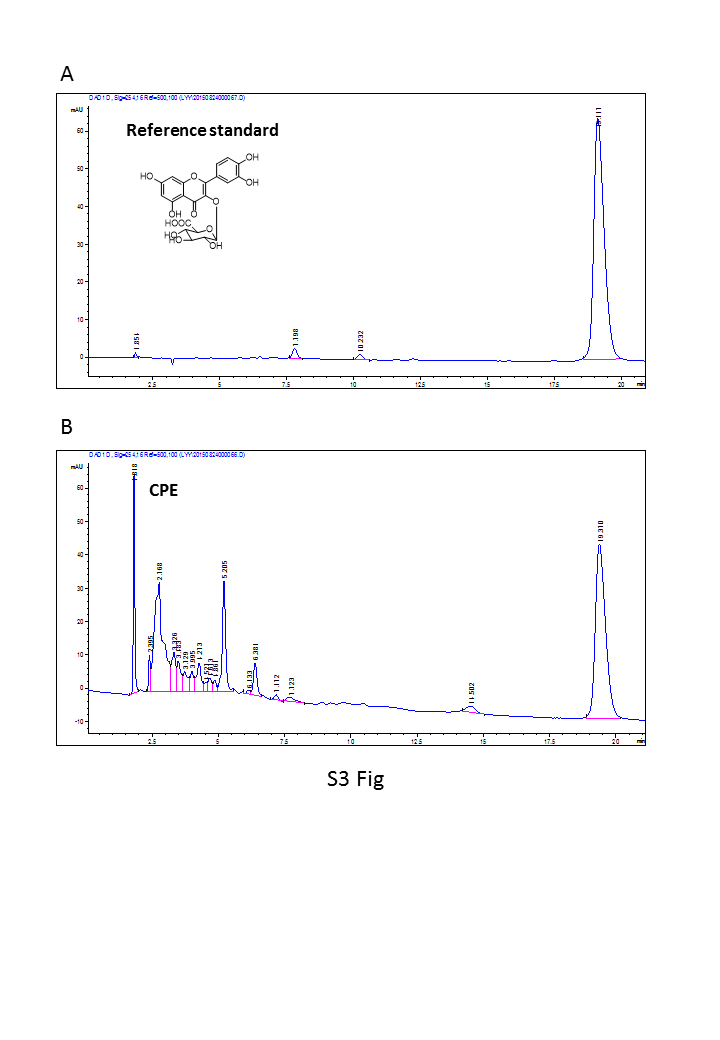

Supplement: S3 Fig — (A) Chromatogram of the reference standards of quercetin-3-O-β-D-glucuronide. (B) Chromatogram of the CPE extract. (TIF) [file pone.0183988.s003.tif]
