# Supplementary material for: Cyclocarya paliurus extract activates insulin signaling via Sirtuin1 in C2C12 myotubes and decreases blood glucose level in mice with impaired insulin secretion
Source: PLoS One. 2017 Aug 31;12(8):e0183988. doi: 10.1371/journal.pone.0183988 (PMC5578601; doi:10.1371/journal.pone.0183988)
Supplement: S1 Text — (DOCX) [file pone.0183988.s004.docx]

**S1 Text. High-performance liquid chromatography (HPLC) analysis method**

**Determination of chlorogenic acid and cryptochlorogenic acid in CPE.**

CPE was dissolved in diluted ethanol (15.0 mg/mL) and sonicated for 30 min. The solution was filtered, and the subsequent filtrate was then collected for analysis. The chlorogenic acid and cryptochlorogenic acid contents in CPE were determined on an Agilent 1200 series high performance liquid chromatograph (California, USA) with a phenomenex C18 (250 mm × 4.6 mm, 5 μm, Phenomenex, USA). The mobile phase was a methanol/0.2% ethylic acid solution (17:83, v/v), and the flow rate was 1.0 mL/min. The analysis was performed at 40°C, and the absorbance at 320 nm was detected. The chlorogenic acid and cryptochlorogenic acid contents were 1.83% and 1.02%, respectively, compared with the standards. Chlorogenic acid (purity >95%) was purchased from the National Institutes for Food and Drug Control (Beijing, China). Cryptochlorogenic acid (purity >95%) was purchased from the Chengdu Pufei De Biotech co., Ltd. (Chengdu, China).

**Determination of quercetin-3-O-β-D-glucuronide in CPE.**

CPE was dissolved in diluted ethanol (15.0 mg/mL) and sonicated for 30 min. The solution was filtered, and the subsequent filtrate was then collected for analysis. The quercetin-3-O-β-D-glucuronide content in CPE was determined on an Agilent 1200 series high performance liquid chromatograph (California, USA) with a phenomenex C18 (250 mm × 4.6 mm, 5 μm, Phenomenex, USA). The mobile phase was a methanol/0.4% ethylic acid solution (45:55, v/v), and the flow rate was 1.0 mL/min. The analysis was performed at 40°C, and the absorbance at 254 nm was detected. The quercetin-3-O-β-D-glucuronide content was 29.12% compared with the standards. quercetin-3-O-β-D-glucuronide (purity >95%) was purchased from the Chengdu Pufei De Biotech co., Ltd. (Chengdu, China).
